# Selections

**Published:** 1892-02

**Authors:** 


### Selections.

---

### CARBOLATE OF CAMPHOR.

This preparation is made (*Therapeutic Gazette*, February, 1891) by adding one part, by weight, of carbolic acid to three parts of camphor, setting aside for twenty-four hours, and straining through gauze. It is a permanent liquid, with a specific gravity of 990. It is thoroughly antiseptic, and possesses unsurpassed germicidal powers. Locally applied to wounds, by means of cotton or gauze, it prevents suppuration. When kept in contact with the skin for several days, it produced an eruption which can, however, be prevented by mixing the liquid with oil. Injected hypodermically, it gives the best results in aborting abscesses or boils and relieving pain.

When placed under the skin, it produces anæsthesia of the surrounding parts, which lasts for several hours. There is some soreness, but no abscess results. The slight smarting felt at first shortly disappears. For hypodermic use, a little ether or pure alcohol should be added to the liquid.

Carbolate of camphor combines readily with alcohol, ether, fixed and essential oils, and petroleum derivatives, but *not* with aqueous solutions or glycerin. It readily dissolves menthol, cocaine, salicylic acid, chloral hydrate, iodoform, and corrosive sublimate.

According to M. B. Cochran, carbolate of camphor gives excellent results when locally applied in inflammations or ulcerations of the tonsils, pharynx, or cervix uteri, and as a dressing in all kinds of wounds, where it readily prevents suppuration, and acts as an antiseptic. As a lubricant in massage it is unsurpassed, especially in contracted muscles and stiffened joints. In herpes and erysipelas, applied with a soft brush, the remedy acts as a specific, relieving the pain and causing a healing process to be set up at once. The writer reports a case of the latter affection in a child three weeks old. There was intense swelling of the face, the eyes had not been seen for two days, and the lips were so swollen that the infant could not suckle. The disease was rapidly advancing to the scalp. A mixture of one part of carbolate of camphor to two parts of olive oil was brushed over the face every three or four hours. The disorder was checked from the first applications. In twenty-four hours a marked change was observed. The child made a final recovery.

Good effects have been observed from the use of the remedy in vaginitis, vulvitis, and pruritus vulvæ; also from its employment in cases of frost-bite. The best results have been obtained by internal administration in the form of capsules, in cases of gastric and intestinal catarrh.—*University Medical Magazine.*

---

### PHYSIOLOGICAL ACTION OF PEROXIDE OF HYDROGEN.

In a preliminary paper on "Peroxide of Hydrogen," which he has continued to study since his original investigations published in 1860, Benjamin Ward Richardson (*The Asclepiad*, first quarter, 1891) adds considerably to our knowledge of a drug which has lately claimed much clinical attention. In his physiological researches he finds that "living cell-structure of every kind, presented to the peroxide solution, if it does not absorb, liberates the oxygen, and seems to distinguish by that phenomenon organic structure that has become cellular, organic, and vital, from that which is not yet cellular or organic, and from that which has passed from the organic and vitalized state to the inorganic and dead." Upon open surfaces meeting with organic material, oxygen is liberated and decomposition produced, a process which is hastened by elevation of temperature and pressure. Again, if introduced within mucous cavities which contain no foreign substance capable of liberating oxygen (*e.g.*, pus), the peroxide is not decomposed. Under these conditions, absorption into the circulation with liberation of oxygen takes place, and if this gas be in too great quantity to be carried away entirely by the red blood-disks, it accumulates in the chambers of the circulation and causes death in the same way as air injected into a vein. A similar result follows injection within the peritoneum. Subcutaneously injected, the peroxide is at once decomposed with the production of temporary emphysema and elevation of temperature, with partial arterialization of venous blood, but without important local trouble resulting. When injected directly into the lung, there is diffusion of oxygen, so that life may be supported for several minutes with respiration entirely cut off. Purulent matter and probably the white blood-corpuscles liberate oxygen very freely from the peroxide; the author inclines to the belief that bacteria may have the same reducing power.—*University Medical Magazine.*

---

### CARBOLIC ACID, CREOLIN, AND LYSOL.

From *Revue d'Hygiene* we extract the following notes on the work of Drs. Remonchemps and Sugg, relative to the comparative disinfecting values of carbolic acid, creolin, and lysol. The last is a comparatively new addition to the list of disinfectants. The result of various studies of creolin were given in the Fifth Annual Report.

The authors used the bacilli and spores of anthrax, of typhoid fever, and of cholera in their provings. On the free spores the agents all act slowly; creolin and lysol act a little more promptly than carbolic acid on cultures in bouillon, but the difference would not amount to much in practice. There is, however, an important element of safety, due to the difference in the poisonous properties of these agents. While it took only .30 gramme of carbolic acid to kill a given weight of rabbit, it required 1.10 of creolin or 2.30 of lysol.

One interesting point brought out is that spots on clothing, soiled with fæcal discharges of typhoid fever and cholera patients, may be completely sterilized in two hours in a one-per-cent solution of any one of these agents cold, but if warmed to 122° F. the sterilization is complete in thirty minutes.

With lysol disinfection of the hands and instruments was accomplished with a five-per-cent solution in five seconds, especially when warm; with a two-and-a-half-per-cent solution ulcers and cavities were disinfected in five minutes.

Lysol is a thick, light-brown liquid, with a specific gravity of 1.042. It is perfectly soluble in water, and forms a clear solution in contradistinction to creolin, which forms a milky opaque emulsion. In washing in a solution of lysol it forms a lather, and thus, while exerting the action of a disinfectant, it plays the part of a soap. It therefore has the advantage of easily and rapidly permeating tissues with which it is brought in contact.—*Sanitary Inspector.*

---

### MERCURIAL STOMATITIS.

Fournier, in his clinical lecture on this subject (*L'Union Médicale*, February 3, 1891), alludes to the fact that the older writers frequently mention this affection as causing deep, even phagedenic ulcerations, which, on healing, bound the jaws firmly together. Abundant hemorrhage accompanied these ulcerations. The teeth were lost and necrosis occurred, even death from exhaustion sometimes following. We do not see such cases now, because we do not believe that the germs of the disease are voided by the salivation produced. Formerly inunctions were much used.

When mercury is given by the mouth, stomatitis is slow to begin and is progressive; it announces its advent by mild symptoms; it does not progress beyond a medium degree of intensity. Stomatitis from inunctions is exactly the opposite: its invasion is sudden; it affects the whole mouth simultaneously; it is very severe.

There are two theories in regard to salivation: one regards it as an inflammation of the salivary glands, the secretion from these irritating the mouth; the other holds that the mercury irritates the mouth and provokes the salivation. Nearly every one believes now that the stomatitis is primary and the salivation secondary. A third theory ascribes the cause to a microbe.

To avoid salivation, be sure that the mouth and teeth are in a healthy state; caution patient to watch for symptoms; keep the mouth clean with a wash of chlorate of potash, and brush the teeth with equal parts of charcoal and cinchona, or cinchona and tannin, with essence of mint to flavor; watch the mouth for any indication of the drug's action; stop the treatment at the first alarm.

When salivation has occurred, touch the eroded points with a crayon of nitrate of silver and order mouth-washes of marsh-mallow and chlorate of potash or alum, two to two-and-a-half per cent. Chlorate of potash may also be given internally. In severe cases chlorate of potash, one and a half to three drachms per day, in sweetened water or gum syrup, will, in a few days, produce a calming effect. During the acute period irrigate with marsh-mallow solution, milk or plain water. Later on potash- or boric-acid washes may be tried and the gums may be painted with cocaine. Light superficial cauterizations may be made over small areas. Sedatives like opium will also be useful in the course of the affection.—*University Medical Magazine.*

---

### THE STRUGGLE AGAINST DISEASE.

Dr. J. Burden Sanderson, in concluding a series of lectures upon the nature of infectious diseases, remarks: "We have learned on the one hand from the experiments of Behring and Kitasato, on the other from those of Büchner and Emmerich, that a substance is contained in the liquor sanguinis that acts in relatively small doses, that is capable of counter-infecting the whole organism, and that by combining antibiotic and antitoxic properties in varying proportions adapts itself to the requirement of the particular infection against which the organism needs to be protected.

"It has been proved that the organism possesses the power of bringing into existence certain chemical agents which are the embodyment of the particular *vis protectrix* required, by which the organism guards itself against some of the most important of the specific infections to which it is exposed.

"To have learned this is to have entered on the beginning of a new progress, to have turned over a new leaf in pathology. It seems to me that it requires no prophetic power to foresee that we are on the very threshold of discoveries in medicine such as will eclipse in their splendor all that have preceded them."—*British Medical Journal.*

